# Ultrasonographic Fetal Growth Charts: An Informatic Approach by Quantitative Analysis of the Impact of Ethnicity on Diagnoses Based on a Preliminary Report on Salentinian Population

**DOI:** 10.1155/2014/386124

**Published:** 2014-06-18

**Authors:** Andrea Tinelli, Mario Alessandro Bochicchio, Lucia Vaira, Antonio Malvasi

**Affiliations:** ^1^Division of Experimental Endoscopic Surgery, Imaging, Technology and Minimally Invasive Therapy, Centre for Interdisciplinary Research Applied to Medicine and Department of Obstetrics and Gynecology, Vito Fazzi Hospital, Piazza Muratore, 73100 Lecce, Italy; ^2^Department of Engineering for Innovation, Software Engineering and Telemedia LAB (SET-Lab), University of Salento, 73100 Lecce, Italy; ^3^Department of Obstetrics and Gynecology, Santa Maria Hospital, 70124 Bari, Italy

## Abstract

Clear guidance on fetal growth assessment is important because of the strong links between growth restriction or macrosomia and adverse perinatal outcome in order to reduce associated morbidity and mortality. Fetal growth curves are extensively adopted to track fetal sizes from the early phases of pregnancy up to delivery. In the literature, a large variety of reference charts are reported but they are mostly up to five decades old. Furthermore, they do not address several variables and factors (e.g., ethnicity, foods, lifestyle, smoke, and physiological and pathological variables), which are very important for a correct evaluation of the fetal well-being. Therefore, currently adopted fetal growth charts are inadequate to support the melting pot of ethnic groups and lifestyles of our society. Customized fetal growth charts are needed to provide an accurate fetal assessment and to avoid unnecessary obstetric interventions at the time of delivery. Starting from the development of a growth chart purposely built for a specific population, in the paper, authors quantify and analyse the impact of the adoption of wrong growth charts on fetal diagnoses. These results come from a preliminary evaluation of a new open service developed to produce personalized growth charts for specific ethnicity, lifestyle, and other parameters.

## 1. Introduction

In current clinical medicine, data coming from medical records and analysis are often used to document diagnostic issue, giving the opportunity of a systematic data meta-analysis to improve patient care and to develop new health-assessment techniques.

Correct assessment of gestational age and fetal growth is essential for optimal obstetric management. For this purpose, ultrasound obstetric scans in pregnancy are routinely used to track fetal growth and to assess fetal health.

Fetal size charts are used to compare the size of a fetus (of known gestational age) with reference data and to compare it on two or more different circumstances.

This can be performed using look-up tables or charts, but, as it is easier to identify any deviation from normal by plotting measurements on charts, the use of charts is recommended and the clinical evidence supports their efficacy.

The detection of a potential abnormal growth by means of intrauterine fetal parameters during pregnancy was proposed by serial US scans by Lubchenco [1], Usher and McLean [2], and Babson and Benda [3], more than five decades ago, and fetal growth assessment is a well-established and mature research field in obstetrics and gynecology [4–6].

Fetal growth charts are compared to statistical data (i.e., reference charts with fetal growth curves, showing average values of biometric parameters as a function of the gestational age) so that clinicians may detect fetal growth associated to fetal intrauterine anomalies [7].

Numerous studies have been conducted to derive reference charts for fetal size. Many, however, have a suboptimal design, using a hospital-based population or having an inappropriate sample size.

The proliferation of further studies on specific subgroups of patients [8–12] and the related proposal of an ever increasing number of reference charts were characterized by a considerable methodological heterogeneity, making them difficult to use for diagnostic purposes.

As a consequence in clinical practice, generic charts are preferred to specific ones or to more complex approaches based on suitable mathematic models [11], because of their feasibility.

Moreover, the World Health Organization (WHO) standards are still commonly based on generic reference charts; they do not differentiate by ethnic origin and are not subject to frequent update, so they are unsuitable to assess the biometric parameters in several cases of practical interest.

To preserve the feasibility of the approach without losing diagnostic power, some authors proposed the adoption of purposely developed software tools (Web Applications, Mobile Application, etc.) allowing us to create customized growth charts [13, 14], based on a regression model fitted to a very large group of newborns.

Medical literature clearly showed its main drawbacks: (A) the number of patients considered in the studies (some thousandth) is low with respect to the total number of newborn per year (about 160 Millions in 2013) in the world; (B) patients considered in the studies are not representative of the variety of anthropometrical factors due to ethnicity, familial aspects, and other relevant internal and external factors; (C) the commonly used growth curves are up to five decades old; they are not updated for the current population and they are not suitable to investigate temporal trends and dynamic aspects in fetal growth curves.

Nevertheless, fetal growth is influenced by a variety of factors, racial, social, and economic among others, as well as specific medical conditions that may preexist or that may develop during pregnancy.

Hence, it is not surprising that fetal biometric parameters show high degree of variation in evaluated population from country to country and from area to area, within the same country. Beyond ethnicity, many other factors affect fetal growth including fetus gender, physiological and pathological variables, maternal height and weight, drug or tobacco exposure, genetic syndromes, congenital anomalies, and placental failure [15–18].

In this context, it is necessary to have personalized charts for fetal growth in order to provide an accurate fetal assessment and to make the presence of false positive and false negative potentially avoidable.

The adoption of wrong reference curves on specific fetuses could cause an incorrect evaluation of fetal biometric parameters, identifying for example cases known in literature as Small for Gestational Age (SGA) or Large for Gestational Age (LGA). So, using personalized growth curves would result in a considerable decline in the rate of a false-positive diagnosis of SGA/LGA.

In this scenario, authors quantify and analyse the impact of the adoption of such wrong growth charts on fetal diagnoses. As initial results, authors show how much different are values and boundaries of certain biometric parameters according to ethnicity. Salentinian population (southeast of Italy) has been analysed and its samples have been compared with the reference curves adopted for Italian [19] and European [20] fetuses.

These preliminary results have been obtained by adopting a new “online service” in charge to develop personalized growth charts, which take into account differences due to ethnicity, lifestyle, familial aspects, and other parameters.

## 2. Material and Methods

The study includes a population of about 500 Italian women undergoing ultrasound examination between the 11th and 41th weeks of gestation, between November 2012 and September 2013.

All pregnant women were enrolled in a previously defined area, southeast of Italy, in the Vito Fazzi Hospital, Italy, and Departments of Obstetrics and Gynecology assessed the investigation.

Gestational age was established by using US imaging during the first visit, at study enrolment. All patients received written and oral information about the study, and they signed the informed consent.

## 3. Data Harvesting Methodology

Before enrolment, authors defined, in the setup study, the inclusion and exclusion criteria Inclusion criteria were: singleton pregnancies, known first day of the Last Menstrual Period (LMP), regular cycle (lasting 28 ±  4 days) The date of the LMP was confirmed with the pregnant woman at the first obstetric visit, and additional information on regularity and duration of the cycle was collected during visit. Cases with low birth weight, preterm delivery, or other prenatal complications were not excluded from analysis. Gestational age was based on the last menstrual period and in all cases adjusted according to the CRL measured in the first trimester ultrasound.

Pregnant women were excluded from analysis if they joined the study after the 24th week of pregnancy, because reliable dating of pregnancy is more difficult as pregnancy proceeds. Bidimensional (2D) US scans were conducted either with a Logic 7 Pro US system (GE-Kretz, Zipf, Austria), an IU 22 xMATRIX US system (Philips Healthcare, Eindhoven, The Netherlands), or a Voluson 730 US system (GE-Kretz, Zipf, Austria) equipped with a 3.8–5.2 MHz transabdominal transducer by resident clinicians well-trained in obstetric US. All machines had a standard US setting of Doppler and grey scale, provided by companies. Measurements of the biparietal diameter (BPD) and head circumference (HC) were obtained from a transverse axial plane of the fetal head showing a central midline echo broken in the anterior third by the cavum of septum pellucidi and demonstrating the anterior and posterior horns of the lateral ventricle. The BPD was measured from the outer margin of the proximal skull to the inner margin of the distal skull. The HC was measured fitting a computer-generated ellipse to include the outer edges of the calvarial margins of the fetal skull. The abdominal circumference (AC) was measured fitting a computer-generated ellipse through a transverse section of the fetal abdomen at the level of the stomach and bifurcation of the main portal vein into its right and left branches. The femur length (FL) was measured in a longitudinal scan where the whole femural diaphysis was seen almost parallel to the transducer and measured from the greater trochanter to the lateral condyle. In the third trimester, particular care was taken not to include the epiphysis.

## 4. Statistical Methods

Each interval of gestational age was centred on a week, so that from 13 weeks and 4 days up to 14 weeks and 3 days has been considered as 14th week.

Statistical analysis has been performed using appropriate packages of R Software (http://www.r-project.org).

The normality of measurements at each week of gestation was assessed using the Shapiro-Wilk test [21], which is one of the most powerful tests to use for the normality assessment, especially for small samples. It tests the null hypothesis that a given sample came from a normally distributed population.

In order to obtain normal ranges for fetal measurements, a multistep procedure based on regression model has been used, according to the recommended methodology for this type of data [22, 23].

Assuming that, at each gestational age, the measurement of interest has a Gaussian distribution with a mean and a standard deviation (SD) and that, in general, both vary smoothly with gestational age, a centile curve has been calculated using the well-known formula:

(1) $$\begin{array}{c} \text{Centile}=\text{mean}+K*\text{SD}, \end{array}$$

where *K* is the corresponding centile of the standard Gaussian distribution (e.g., determination of 10th and 90th centile curves requires that *K* = ±1.28), mean is the mean, and SD is the standard deviation of the mean of the fetal measurements for each gestational age.

The mean has been estimated by the fitted values from an appropriate polynomial regression curve of the measurement of interest on gestational age.

Several curve-fitting and smoothing techniques have been tested for the mean estimation of the different biometric parameters and the goodness of fit for each regression model has been carefully assessed. The polynomial model that better satisfies the experimental data is the cubic one, since it better fulfils the fractional polynomial and the logarithmic transformations.

The adopted equation is

(2) $$\begin{array}{c} y=a+\left(b*\text{GA}\right)+\left(c*\text{G}{\text{A}}^{2}\right)+\left(d*{\text{GA}}^{3}\right). \end{array}$$

When the measurement has approximately a Gaussian distribution, the fitted values following regression of the “scaled absolute residuals” on age are estimate of the SD curve. These residuals are the differences between the measurements and the estimated curve for the mean with the sign removed and multiplied by a corrective constant equal to $\sqrt{\left(\pi /2\right)}=1.253$.

Generally, if the scaled absolute residuals appear to show no trend with gestational age, the SD is estimated as the standard deviation of the unscaled residuals (measurements minus the estimated mean curve). If there is a trend, then polynomial regression analysis is needed to estimate an appropriate curve in the same way of the mean.

For BPD, HC, and AC biometric parameters, the residuals were regressed on gestational ages by using a linear model in the form of

(3) $$\begin{array}{c} y_{\text{BPD},\text{HC},\mathrm{AC}}=a+\left(b*\text{GA}\right), \end{array}$$

While, considering the FL parameter, the quadratic regression seems to better fulfil the linear one. The adopted equation is

(4) $$\begin{array}{c} y_{\text{FL}}=a+\left(b*\text{GA}\right)+\left(c*{\text{GA}}^{2}\right). \end{array}$$

Finally, these predictive mean and SD equations allow calculating any required centile, replacing the value in the centile formula.

## 5. Results

Full biometric measurements (AC, BPD, FL, and HC) were obtained for about 500 fetuses.

Data analysis showed that neither the use of fractional polynomials (the greatest power of the polynomials being 3) nor the logarithmic transformation improved the fitting of the curves. Therefore, the data were kept in their original scale. The best-fitted regression model to describe the relationships between HC, AC, BPD, and FL and gestational age was a cubic one, whereas other studies proved that a simple quadratic model fitted BPD and FL [24].

Models fitting the SD were straight lines for BPD, HC, and AC and quadratic line for FL.

To choose the best fitting model, we have taken into consideration primarily the *R* ^2^ index (which is the linear determination index: in the ideal case its value should be equal to 1; in real cases it is near to 1 if the interpolating curve is a good approximation of the real data set) but the value of *R* ^2^ alone is not the only factor that we have considered in choosing the best model. Other factors we have considered include the validity and the effectiveness of the model.

There will be an improvement in fit as higher-order terms are added, but because these terms are not theoretically justified, the improvement will be sample-specific.

Unless the sample is very small, the fits of higher-order polynomials are unlikely to be very different from those of a quadratic over the main part of the data range.

Consider that, for example, the *R* ^2^ for the quadratic specification of BPD parameter is 0.98081 and for the cubic and quartic curves it is 0.98229 and 0.98242, relatively small improvements.

**Figure 1:**
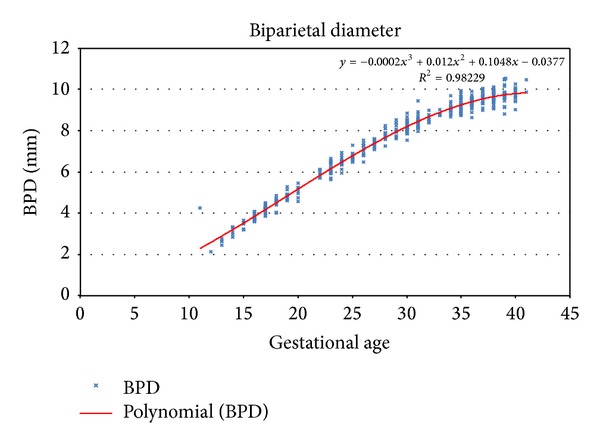
Third order polynomial regression for biparietal diameter.

**Figure 2:**
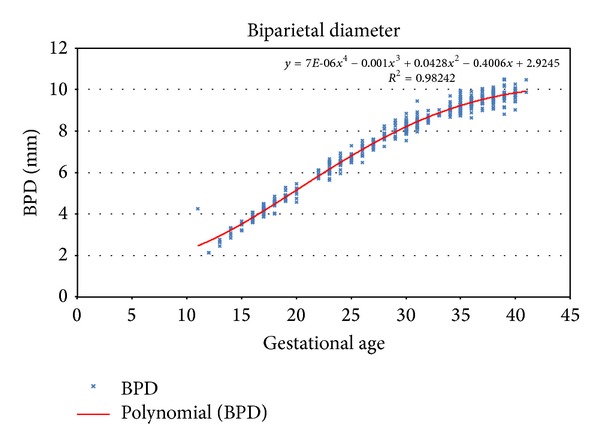
Fourth order polynomial regression for biparietal diameter.

Further, the cubic and quartic curves both exhibit implausible strange twists at the extremities (Figures 1 and 2).

**Figure 3:**
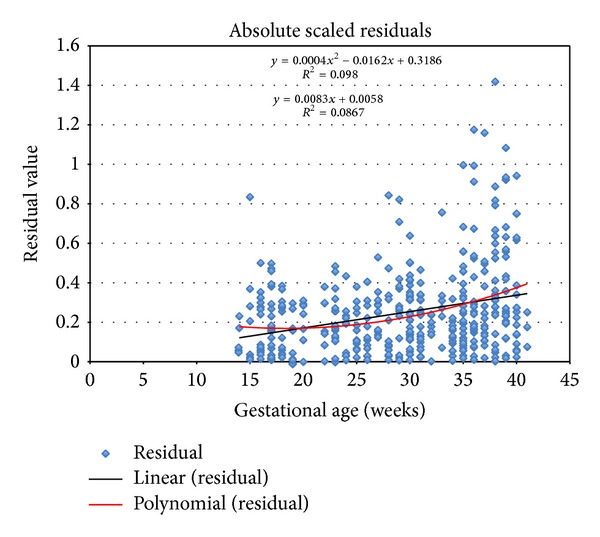
Absolute scaled residuals for FL measurement.

The scatter of absolute residuals from the regression for estimation of the standard deviation of femur length as a function of gestational age is shown in Figure 3.

The corresponding regression equations, with the respective *R* ^2^ index for the mean and the standard deviation, are illustrated in Table 1.

Table 1 shows regression equations for the mean and the standard deviation of AC, BPD, FL, and HC.

The relevant centile (5th, 10th, 50th, 90th, and 95th), representing, respectively, the HC, the BPD, the A,C and the FL, are reported in Tables 2, 3, 4, and 5. In each table, it is also indicated that the sample number, the mean, and the standard deviation are related to each gestational week.

## 6. Discussion

In order to validate the system, authors have performed an initial technical test with a growth curve simulator able to respect the mean and the standard deviation that characterize the Gaussian distribution for a specific patient age. The generated data allowed authors to prove the correctness of the elaboration of the fetal growth curves model.

After this preliminary analysis, authors have performed a test on the field considering about 500 US pictures related to Italian women undergoing ultrasound examination between the 11th and 41th weeks of gestation at Vito Fazzi Hospital, Lecce, between November 2012 and September 2013. Measurements of biparietal diameter (BPD), head circumference (HC), abdominal circumference (AC), and femur length (FL) were obtained during the clinical practice.

The obtained curves were then compared with those developed by Giorlandino et al. [19] as reference growth curves for the Italian population and those developed by Johnsen et al. [20] as reference growth curves for the European population, in order to verify possible differences due to statistic methodology, selection criteria, or, possibly, true genetic variability of the studied population.

The AC and HC biometric parameters seem to follow more or less the same Italian and European trend according to the gestational age. In fact, no significant differences were observed in the values measured during the different growth stages. Considering the BPD and the FL parameters, instead, they present a little variability.

**Figure 4:**
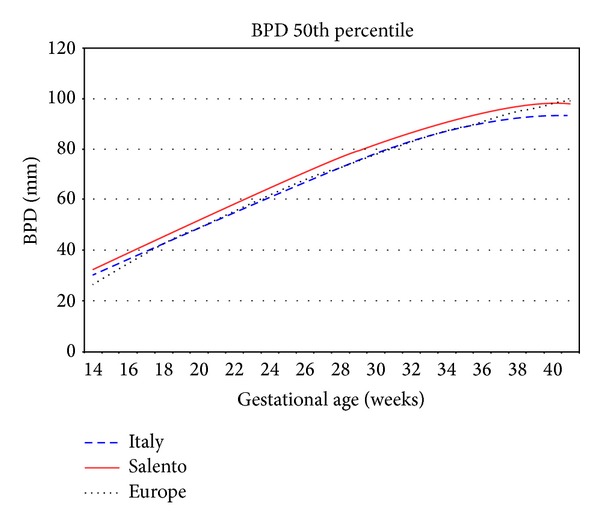
Biparietal diameter 50th percentile Salento versus Italy versus Europe.

**Figure 5:**
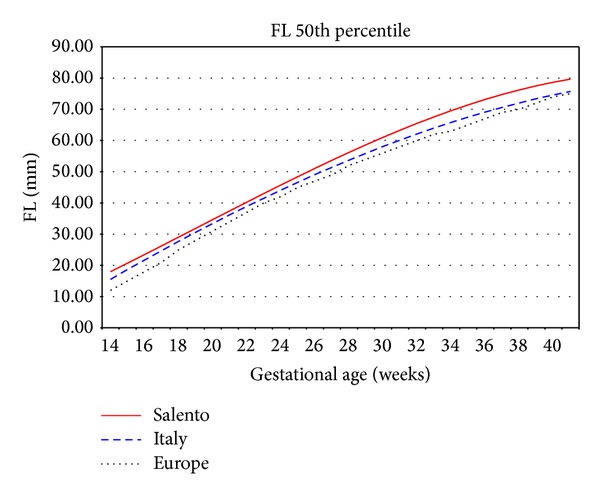
Femur length 50th percentile Salento versus Italy versus Europe.

As shown in Figures 4 and 5, the Salentinian BPD values are always up for about 6 mm and FL ones are always greater than 7 mm.

This variability may be better presented by means of scatterplot of Salentinian samples overlapped with the centile curves to verify the amount and the density of the samples that are outside the considered range.

**Figure 6:**
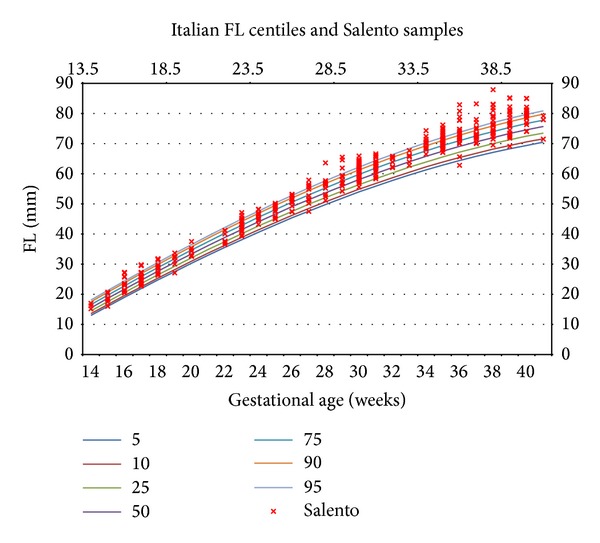
Femur length: Italian centiles and Salentinian samples.

Considering the Italian reference centile curves depicted in Figure 6, which represent, respectively, the 5th, 10th, 25th, 50th, 75th, 90th, and 95th, the Salentinian samples are always above the upper limit, especially in the last weeks of gestation.

Samples above the 95th centile are traditionally used to define large for gestational age (LGA), and the usage of such Italian reference curves on a Salentinian fetus could lead to misdiagnosis.

To examine in a quick way one or more sets of data graphically, box plots can be used. They can be useful to indicate the degree of dispersion (spread) and skewness in the data and to identify outliers. Each plot depicts the five-number summaries for each biometric parameter, namely, the minimum and maximum values, the upper (Q3) and lower (Q1) quartiles, and the median.

The variability present in the FL parameter can be also observed in this kind of graph, which considers more population groups.

**Figure 7:**
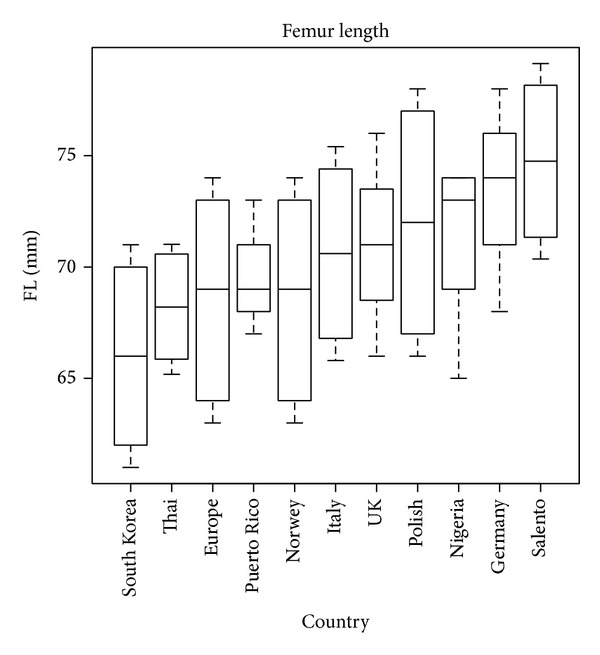
Femur length box plot.

As can be seen in Figure 7, an average length that is similar to that of Germany characterizes the Salentinian femur. Its maximum value is rather close to that of the UK.

This variability has to be medically investigated since it can be due to several reasons: equipment or measurement errors, genetic variability of the analysed population, racial factors, and so on.

In any case, the measured variability is useful to demonstrate the effectiveness of the proposed approach.

The complete set of curves obtained from the mentioned dataset and the complete description of the mathematical procedure adopted for the analysis are published and described at http://www.fpgt.unisalento.it/FPGT/Projects/scientificFoundations.php.

In order to quantify the impact of the adoption of wrong growth charts on fetal diagnoses, authors have analysed the samples' trend for each biometric parameter and have then compared it with the Italian and European standard.

Authors found significant differences between Salentinian FL growth plots and those reported by Giorlandino et al. [19] for Italy and Johnsen et al. [20] for Europe.

From Tables 6, 7, 8, and 9, we describe this difference, representing the sample number and the percentage value for each biometric parameters (BPD, HC, AC, and FL) which exceed the upper limit (95th centile) and the lower one (5th centile) considering the Italian and European reference curves.

## 7. Conclusions

The fetal growth assessment is a relevant problem, since it concerns about 160 ML of newborns per year. The population reshuffling and the increased mobility of families push for a new assessment approach based on dynamic and individualized fetal growth curves.

The importance of the growth curves is proven by the fact that they are commonly used in neonatal units today. They serve as standard references to classify neonates as SGA, LGA, and AGA. In order to evaluate the applicability of these standards to current patients, we compared data accumulated in our research data system to determine whether our patients were categorized appropriately.

Our findings require that we should carefully reexamine the appropriateness of continued use of currently adopted reference growth curves to classify neonates SGA, LGA, and AGA.

In fact, considering, for example, the femur length parameter, Salentinian fetuses present bigger values with respect to those of Italy (26% of Salentinian samples are above the 95th centile) and Europe (46% of Salentinian samples are above the 95th centile).

This is a preliminary approach, which does not represent the development and publication of new reference curves for Salentinian population but rather represents the introduction of a new method to construct the fetal growth curves which has to take into consideration several information about ethnicity, foods, lifestyle, drugs assumption, and other internal or external factors influencing growth.

## Figures and Tables

**Table 1:** Regression equations for the mean and the standard deviation of AC, BPD, FL, and HC.

Fetal parameter	Regression equations	*R* ^2^ (%)
Abdominal circumference (AC)		
Mean	2,2783 − 0,07057 GA + 0,05214 GA^2^ − 0,0007706 GA^3^	94
SD	0,0284 + 0,354 ∗ GA	
Biparietal diameter (BPD)		
Mean	−0,0377 + 0,10476 GA + 0,012021 GA^2^ − 0,0002124 GA^3^	98
SD	0,0762 + 0,0042 ∗ GA	
Femur length (FL)		
Mean	−1,2394 + 0,13735 GA + 0,007537 GA^2^ − 0,000132 GA^3^	98
SD	0,3186 − 0,0162 ∗ GA + 0,0004 ∗ GA^2^	98
Head circumference (HC)		
Mean	−9,8027 + 1,4258 GA + 0,006556 GA^2^ − 0,0003765 GA^3^	98
SD	0,453 + 0,0102 ∗ GA	

**Table 2:** Fitted centiles of head circumference (mm).

GA (weeks)	Sample size (*n*)	Mean HC (mm)	SD	Percentile				
				5th	10th	50th	90th	95th
14	5	104,10	0,60	94,31	96,48	104,10	111,73	113,89
15	7	117,89	0,61	107,93	110,13	117,89	125,64	127,84
16	17	131,46	0,62	121,34	123,57	131,46	139,35	141,59
17	30	144,81	0,63	134,52	136,79	144,81	152,83	155,10
18	13	157,90	0,64	147,44	149,75	157,90	166,05	168,36
19	12	170,72	0,65	160,09	162,44	170,72	179,00	181,35
20	7	183,24	0,66	172,44	174,83	183,24	191,65	194,03
21	—	195,44	0,67	184,47	186,89	195,44	203,98	206,40
22	7	207,29	0,68	196,16	198,62	207,29	215,96	218,42
23	20	218,78	0,69	207,48	209,98	218,78	227,58	230,08
24	13	229,88	0,70	218,41	220,95	229,88	238,81	241,35
25	13	240,57	0,71	228,94	231,51	240,57	249,63	252,20
26	14	250,83	0,72	239,02	241,63	250,83	260,02	262,63
27	12	260,63	0,73	248,66	251,30	260,63	269,95	272,60
28	12	269,95	0,74	257,81	260,49	269,95	279,40	282,08
29	13	278,77	0,75	266,46	269,18	278,77	288,35	291,07
30	42	287,06	0,76	274,59	277,34	287,06	296,78	299,54
31	23	294,81	0,77	282,17	284,96	294,81	304,66	307,45
32	8	301,99	0,78	289,18	292,01	301,99	311,97	314,80
33	12	308,58	0,79	295,60	298,47	308,58	318,69	321,56
34	15	314,55	0,80	301,41	304,31	314,55	324,79	327,70
35	32	319,89	0,81	306,58	309,52	319,89	330,26	333,20
36	27	324,57	0,82	311,09	314,06	324,57	335,07	338,05
37	19	328,56	0,83	314,91	317,93	328,56	339,20	342,21
38	28	331,85	0,84	318,04	321,09	331,85	342,62	345,67
39	25	334,42	0,85	320,43	323,52	334,42	345,31	348,40
40	15	336,23	0,86	322,08	325,20	336,23	347,25	350,38
41	3	337,27	0,87	322,95	326,11	337,27	348,43	351,59

**Table 3:** Fitted centiles of biparietal diameter (mm).

GA (weeks)	Sample size (*n*)	Mean BPD (mm)	SD	Percentile				
				5th	10th	50th	90th	95th
14	5	32,02	0,14	29,80	30,29	32,02	33,75	34,24
15	7	35,22	0,14	32,93	33,43	35,22	37,00	37,51
16	17	38,46	0,14	36,10	36,62	38,46	40,30	40,82
17	30	41,74	0,15	39,31	39,85	41,74	43,63	44,17
18	14	45,04	0,15	42,54	43,10	45,04	46,99	47,54
19	14	48,35	0,16	45,79	46,36	48,35	50,35	50,92
20	7	51,67	0,16	49,03	49,61	51,67	53,72	54,30
21	—	54,96	0,16	52,26	52,86	54,96	57,07	57,67
22	7	58,24	0,17	55,46	56,07	58,24	60,40	61,01
23	20	61,47	0,17	58,62	59,25	61,47	63,68	64,31
24	13	64,64	0,18	61,73	62,38	64,64	66,91	67,56
25	13	67,76	0,18	64,78	65,43	67,76	70,08	70,74
26	13	70,79	0,19	67,74	68,42	70,79	73,17	73,84
27	13	73,73	0,19	70,62	71,30	73,73	76,16	76,85
28	12	76,57	0,19	73,39	74,09	76,57	79,06	79,76
29	13	79,30	0,20	76,04	76,76	79,30	81,84	82,55
30	43	81,89	0,20	78,57	79,30	81,89	84,48	85,22
31	22	84,34	0,21	80,95	81,70	84,34	86,99	87,74
32	8	86,64	0,21	83,18	83,94	86,64	89,34	90,11
33	6	88,77	0,21	85,24	86,02	88,77	91,53	92,31
34	15	90,72	0,22	87,12	87,92	90,72	93,53	94,32
35	33	92,48	0,22	88,81	89,62	92,48	95,34	96,15
36	30	94,03	0,23	90,29	91,12	94,03	96,95	97,77
37	18	95,36	0,23	91,56	92,40	95,36	98,33	99,17
38	30	96,47	0,24	92,59	93,44	96,47	99,49	100,35
39	26	97,33	0,24	93,38	94,25	97,33	100,40	101,27
40	17	97,93	0,24	93,91	94,80	97,93	101,06	101,94
41	2	98,26	0,25	94,17	95,08	98,26	101,44	102,35

**Table 4:** Fitted centiles of abdominal circumference (mm).

GA (weeks)	Sample size (*n*)	Mean AC (mm)	SD	Percentile				
				5th	10th	50th	90th	95th
14	5	93,95	0,52	85,33	87,24	93,95	100,67	102,57
15	6	103,50	0,56	94,30	96,34	103,50	110,67	112,71
16	17	113,41	0,59	103,62	105,78	113,41	121,03	123,19
17	29	123,61	0,63	113,24	115,53	123,61	131,69	133,98
18	13	134,07	0,67	123,12	125,54	134,07	142,60	145,02
19	12	144,74	0,70	133,21	135,76	144,74	153,73	156,28
20	7	155,58	0,74	143,47	146,14	155,58	165,02	167,69
21	—	166,54	0,77	153,84	156,64	166,54	176,43	179,23
22	7	177,56	0,81	164,28	167,22	177,56	187,91	190,84
23	19	188,61	0,84	174,75	177,81	188,61	199,41	202,47
24	13	199,64	0,88	185,20	188,39	199,64	210,90	214,09
25	12	210,61	0,91	195,58	198,90	210,61	222,32	225,63
26	14	221,46	0,95	205,85	209,30	221,46	233,62	237,07
27	12	232,15	0,98	215,96	219,54	232,15	244,77	248,34
28	12	242,64	1,02	225,87	229,57	242,64	255,71	259,41
29	13	252,87	1,06	235,52	239,35	252,87	266,39	270,23
30	43	262,81	1,09	244,87	248,84	262,81	276,78	280,75
31	22	272,40	1,13	253,88	257,97	272,40	286,83	290,92
32	7	281,60	1,16	262,50	266,72	281,60	296,49	300,70
33	6	290,37	1,20	270,69	275,03	290,37	305,70	310,05
34	14	298,65	1,23	278,39	282,86	298,65	314,44	318,92
35	33	306,40	1,27	285,56	290,16	306,40	322,65	327,25
36	25	313,58	1,30	292,15	296,88	313,58	330,28	335,01
37	19	320,14	1,34	298,12	302,99	320,14	337,29	342,15
38	29	326,02	1,37	303,43	308,42	326,02	343,63	348,62
39	26	331,20	1,41	308,02	313,14	331,20	349,26	354,37
40	18	335,61	1,44	311,85	317,10	335,61	354,12	359,37
41	3	339,22	1,48	314,88	320,25	339,22	358,18	363,56

**Table 5:** Fitted centiles of femur length (mm).

GA (weeks)	Sample size (*n*)	Mean AC (mm)	SD	Percentile				
				5th	10th	50th	90th	95th
14	5	17,99	0,17	15,19	15,80	17,99	20,17	20,79
15	7	20,71	0,17	17,99	18,59	20,71	22,83	23,44
16	17	23,47	0,16	20,81	21,40	23,47	25,54	26,13
17	29	26,25	0,16	23,64	24,22	26,25	28,29	28,86
18	13	29,05	0,16	26,47	27,04	29,05	31,06	31,63
19	12	31,86	0,16	29,30	29,87	31,86	33,85	34,41
20	7	34,66	0,15	32,12	32,68	34,66	36,65	37,21
21	—	37,46	0,15	34,92	35,48	37,46	39,45	40,01
22	7	40,25	0,16	37,68	38,25	40,25	42,24	42,81
23	21	43,01	0,16	40,41	40,99	43,01	45,03	45,60
24	12	45,74	0,16	43,10	43,68	45,74	47,79	48,37
25	13	48,42	0,16	45,73	46,33	48,42	50,52	51,12
26	14	51,07	0,17	48,31	48,92	51,07	53,22	53,83
27	12	53,65	0,17	50,81	51,44	53,65	55,87	56,50
28	12	56,18	0,18	53,24	53,89	56,18	58,47	59,12
29	13	58,63	0,19	55,58	56,26	58,63	61,00	61,68
30	42	61,00	0,19	57,84	58,54	61,00	63,47	64,17
31	21	63,29	0,20	59,99	60,72	63,29	65,86	66,59
32	7	65,48	0,21	62,03	62,79	65,48	68,17	68,93
33	6	67,57	0,22	63,96	64,76	67,57	70,39	71,18
34	14	69,55	0,23	65,76	66,60	69,55	72,50	73,34
35	31	71,41	0,24	67,44	68,32	71,41	74,51	75,39
36	25	73,15	0,25	68,97	69,89	73,15	76,40	77,32
37	19	74,75	0,27	70,36	71,33	74,75	78,16	79,13
38	29	76,20	0,28	71,59	72,61	76,20	79,80	80,82
39	28	77,51	0,30	72,65	73,73	77,51	81,29	82,36
40	18	78,66	0,31	73,55	74,68	78,66	82,64	83,77
41	3	79,64	0,33	74,27	75,45	79,64	83,83	85,02

**Table 6:** Synthetic values for biparietal diameter (BPD).

	Biparietal diameter					
	Salento		Italy		Europe	
	Samples	%	Samples	%	Samples	%
>95th centile	49/448	10,93%	99/448	22%	69/448	15,40%
<5th centile	41/448	9,15%	0/448	0%	2/448	0,44%

**Table 7:** Synthetic values for abdominal circumference (AC).

	Abdominal circumference					
	Salento		Italy		Europe	
	Samples	%	Samples	%	Samples	%
>95th centile	34/436	7,79%	7/436	1,60%	20/436	4,58%
<5th centile	21/436	4,81%	13/436	2,98%	13/436	2,98%

**Table 8:** Synthetic values for head circumference (HC).

	Head circumference					
	Salento		Italy		Europe	
	Samples	%	Samples	%	Samples	%
>95th centile	17/444	3,82%	4/444	0,90%	65/444	14,60%
<5th centile	23/444	5,18%	20/444	4,50%	1/444	0,22%

**Table 9:** Synthetic values for femur length (FL).

	Femur length					
	Salento		Italy		Europe	
	Samples	%	Samples	%	Samples	%
>95th centile	42/437	9,61%	115/437	26,00%	202/437	46%
<5th centile	30/437	6,86%	1/437	0,20%	0/437	0%
